# Oxygen Cost of Walking in Persons with Multiple Sclerosis: Disability Matters, but Why?

**DOI:** 10.1155/2014/162765

**Published:** 2014-03-06

**Authors:** Brian M. Sandroff, Rachel E. Klaren, Lara A. Pilutti, Robert W. Motl

**Affiliations:** Department of Kinesiology and Community Health, University of Illinois at Urbana-Champaign, 233 Freer Hall, 906 South Goodwin Avenue, Urbana, IL 61801, USA

## Abstract

*Background.* The oxygen cost (O_2_ cost) of walking is elevated in persons with MS, particularly as a function of increasing disability status. *Objective.* The current study examined symptomatic (i.e., fatigue, pain, anxiety, and depression) and gait (i.e., velocity, cadence, and step length) variables that might explain why disability status is associated with O_2_ cost of walking in persons with MS. *Materials and Methods.* 82 participants completed the Patient-Determined Disease Steps, Fatigue Severity Scale, McGill Pain Questionnaire, and Hospital Anxiety and Depression Scale and undertook 2 trials of walking on a GAITRite electronic walkway. Participants then completed a six-minute walk test with concurrent assessment of expired gases for quantifying oxygen consumption and O_2_ cost of walking. *Results.* Disability (*r* = 0.55) as well as fatigue (*r* = 0.22), gait velocity (*r* = −0.62), cadence (*r* = −0.73), and step length (*r* = −0.53) were associated with the O_2_ cost of walking. Cadence (*β* = −0.67), but not step length (*β* = −0.14) or fatigue (*β* = −0.10), explained the association between disability and the O_2_ cost of walking. *Conclusions.* These results highlight cadence as a target of rehabilitation for increasing metabolic efficiency during walking among those with MS, particularly as a function of worsening disability.

## 1. Introduction

The impairment of walking is a hallmark feature of neurological diseases such as multiple sclerosis (MS) [1, 2]. For example, there is evidence that persons with MS expend more energy than controls without MS when performing comparable walking assessments (e.g., 6 minutes of walking at 3 mph) [3, 4]. This increase in energy expenditure can be expressed in terms of the oxygen cost (O_2_ cost) of walking. The O_2_ cost of walking is defined as the amount of oxygen consumed per kilogram of body weight over a distance traveled (i.e., mL/kg/m). Conceptually, the O_2_ cost of walking reflects the total energy required for over-ground ambulation and can change as a function of reduced distance traveled for the same amount of energy expended or as a function of increased energy expenditure for traveling the same distance. The O_2_ cost of walking, therefore, represents a physiological marker of walking impairment that reflects the contribution of pathologic gait abnormalities and possibly symptomatic manifestations caused by neurological disability [5].

Of note, there is preliminary evidence that the O_2_ cost of walking is associated with disability status and perhaps other disease-related consequences in persons with mild MS. For example, one study reported that those with worse disability demonstrated a higher O_2_ cost of walking during 6-minute bouts of walking on a treadmill or over-ground in samples of 18 and 24 persons with MS, respectively [6]. Another study reported that the O_2_ cost of walking on a treadmill was associated with spatiotemporal parameters of gait (e.g., velocity, stride length, and double limb support) and symptomatic fatigue in 44 persons with MS who had minimal disability [7]; other research has reported a nonsignificant correlation between the O_2_ cost of walking and symptomatic fatigue [8]. One limitation of those previous studies is the inclusion of persons with relatively mild MS-related disability; this limits our understanding of the association between disability and O_2_ cost of walking across a broader range of mobility impairment in MS. There further is minimal research on variables that explain the association between disability and O_2_ cost of walking; this limits our understanding of targets for possibly reducing the O_2_ cost of walking across the mobility-disability spectrum in MS.

The current study examined potential symptomatic (i.e., fatigue, pain, anxiety, and depression) and gait (i.e., velocity, cadence, and step length) variables as factors that might explain the association between disability status and the O_2_ cost of walking in a relatively large sample of 82 persons with MS who had a broad range of disability. We expected that disability status would correlate with O_2_ cost of walking and that the association would be accounted for by gait parameters and symptomatic variables. Such an examination is important for (a) further understanding the O_2_ cost of walking as a function of neurological disability status in persons with MS and (b) identifying targets of interventions for reducing the O_2_ cost of walking in this population.

## 2. Materials and Methods

### 2.1. Participants

The data were secondary outcomes from the baseline testing session of a behavioral intervention for increasing physical activity in persons with MS [9]. Participants were contacted (a) by mail through a flyer that was sent to patients in North American Research Committee on Multiple Sclerosis (NARCOMS) registry or (b) by e-mail through a flyer that was sent to participants in a database from previous studies conducted in our laboratory over the past five years. There were 511 participants who initially expressed interest and who were contacted via phone by the project coordinator. After explaining the study protocol, the project coordinator undertook screening for inclusion with 230 individuals who remained interested. The inclusion criteria involved (a) having a definite diagnosis of MS (i.e., physician's verification of MS diagnosis), (b) being relapse-free for the past 30 days, (c) being able to walk with or without an assistive device (i.e., cane, crutch, or walker) for collection of O_2_ cost of walking and gait outcomes, (d) being between 18 and 64 years of age, (e) being willing and able to travel to our laboratory to complete the walking assessments, (f) participating in less than 3 days per week of physical activity behavior, (g) having a low risk for contraindications of physical activity based on no more than a single “yes” response on the Physical Activity Readiness Questionnaire (PAR-Q) [10], and (h) being able to provide a physician's approval for participation in the study. Of the 230 persons who were screened, 106 did not meet inclusion criteria, with the primary reasons being too physically active (*n* = 57) or unwilling to travel to our laboratory (*n* = 23); 39 additional persons did not provide physician's approval; and 3 cancelled the testing session due to scheduling conflicts. This resulted in a final sample of 82 persons with MS who were enrolled in this study.

### 2.2. Primary Measures

#### 2.2.1. Oxygen Cost of Walking

Oxygen consumption (VO_2_) was measured breath-by-breath using a commercially available portable metabolic unit (K4b2 Cosmed, Italy) during a standard 6-minute walk (6 MW) test that was performed over-ground in a hallway with two 180-degree turns. The O_2_ and CO_2_ analyzers of the portable metabolic unit were calibrated using known concentrations of gases, and the flow-meter was calibrated using a 3 L syringe (Hans Rudolph, Kansas City, MO). We measured VO_2_ (mL/kg/min) as 30-second averages for 1 minute both before the 6 MW (i.e., resting VO_2_) and over the entire 6 MW. We further measured total distance traveled (m) using a measuring wheel (Stanley MW50, New Briton, CT). We determined net steady-state VO_2_ by (a) calculating steady-state VO_2_ as average VO_2_ values across the final 3 minutes of the 6 MW (i.e., steady-state VO_2_; minutes 4–6) and (b) subtracting resting-state VO_2_ values for the 1 minute prior to the 6 MW. The O_2_ cost of walking was then computed by dividing net steady-state VO_2_ (mL/kg/min) by walking speed during the 6MW (m/min) [5]; this resulted in O_2_ cost of walking expressed as mL/kg/m.

#### 2.2.2. Disability Status

The Patient Determined Disease Steps (PDDS) scale [11] was included as a self-report measure of disability status. The PDDS was developed as an inexpensive surrogate for the Expanded Disability Status Scale (EDSS) [12] and contains a single item for measuring self-reported neurological impairment on an ordinal scale, ranging from 0 (normal) to 8 (bedridden). This scale has been validated in MS based on a strong correlation with a neurologist-administered EDSS (*ρ* = 0.78) and comparable strong correlations between the PDDS and EDSS with other markers of ambulatory status (e.g., Multiple Sclerosis Walking Scale-12, T25FW, 6MW distance) [13].

#### 2.2.3. Symptoms

Fatigue was measured with the 9-item Fatigue Severity Scale (FSS) [14]. Pain was measured using the 15-item, short form of the McGill Pain Questionnaire (SF-MPQ) [15]. Symptoms of anxiety and depression were measured using the Hospital Anxiety and Depression Scale (HADS) [16]. These scales have all been included in previous research involving persons with MS [17–20].

#### 2.2.4. Gait Parameters

Participants completed two trials of walking on a 16-foot GAITRite (CIR Systems, Inc.) electronic walkway at a self-selected pace for measuring gait outcomes. We recorded velocity (cm/sec), cadence (steps/min), and step length (cm) among other variables provided by the GAITRite. These variables were selected based on previously reported differences between persons with MS and healthy controls [21] and associations with disability status [22] and 6 MW distance [23] in samples of persons with MS. We averaged the recorded values per variable across both trials.

### 2.3. Protocol

The protocol was approved by a University Institutional Review Board and all participants provided written informed consent. The protocol included a single session for collecting all data on the University of Illinois at Urbana-Champaign campus. All participants initially completed a battery of questionnaires including a demographics inventory, PDDS, FSS, SF-MPQ, and HADS. Participants then completed two trials on the GAITRite, followed by 10 minutes of seated rest. Participants were fitted with the portable metabolic system during the seated rest. Once wearing the system and after ensuring normal, resting metabolic functioning, participants were given standardized instructions for undertaking the 6 MW and then completed the 6 MW test. All participants were remunerated $50 for completing the session.

### 2.4. Data Analysis

All data were analyzed in SPSS version 21.0 (SPSS Inc., Chicago, IL). Descriptive statistics are presented as means (standard deviations), unless otherwise noted. We first examined the data for outliers using visual inspection of scatter plots and distributions of data; persons with data points that were 3.29 standard deviations above the mean on any measure were considered extreme cases and subsequently Winsorized [24]. Missing data were replaced with the group mean [24]. We then performed bivariate Pearson product-moment correlations (*r*) among disability status, symptoms, gait variables, and the O_2_ cost of walking. Values for correlation coefficients of 0.1, 0.3, and 0.5 were interpreted as small, moderate, and large, respectively [25]. We performed hierarchical linear regression with direct entry to examine if symptomatic/gait variables explained the association between disability status and the O_2_ cost of walking. This was undertaken by regressing the O_2_ cost of walking on disability status in Step 1 and then adding symptomatic and gait variables that correlated with both disability and O_2_ cost of walking in Step 2. We compared the **β*-*coefficient for disability status between Step 1 and Step 2 to examine if symptomatic and gait variables accounted for the association between disability and O_2_ cost of walking. We further examined the **β*-*coefficients for the symptomatic and gait variables to identify the independent contributions for explaining differences in the O_2_ cost of walking.

## 3. Results

### 3.1. Demographic and Clinical Characteristics

The sample included 82 persons with a definite diagnosis of MS. The sample was mostly female (*n* = 63/82; 76.8%), with an average age of 49.1 (SD = 9.0) years, height of 169.0 (SD = 8.5) centimeters, and weight of 80.3 (SD = 21.7) kilograms. 64 participants reported having relapsing-remitting MS (78.0%), and 18 reported having a progressive clinical course (22.0%). The mean duration of MS was 11.9 (SD = 8.1) years, and the median PDDS score was 3 (range = 0–6) indicating moderate disability (i.e., the onset of gait impairment) with a range in mobility disability between normal and bilateral support.

### 3.2. Symptomatic, Gait, and Oxygen Cost of Walking Characteristics

**Figure 1:**
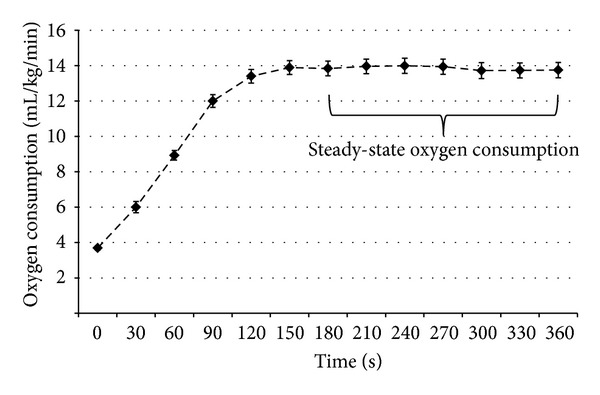
Oxygen consumption over a six-minute walk test in 82 persons with multiple sclerosis. VO_2_  = oxygen consumption (mL/kg/min).

Symptomatic and gait outcomes are reported in Table 1. Collectively, the current sample demonstrated elevated fatigue, pain, anxiety, and depression typical of persons with MS [17–20]. The sample further had similar gait characteristics compared with other samples of persons with MS [26]. Average resting-state VO_2_ was 3.7 (SD = 1.1) mL/kg/min, and this is expected as one MET (i.e., one metabolic equivalent; 3.5 mL/kg/min) is generally considered to be resting-state and our resting VO_2_ value corresponds with 1.1 METs.  Figure 1 depicts VO_2_ in 30-second intervals over the course of 6 MW. This is indicative of steady-state VO_2_ occurring at approximately 180 seconds into the 6 MW; the average steady-state VO_2_ was 13.9 (SD = 3.8) mL/kg/min and this would be considered moderate-intensity walking as it approximated 4 METS [27]. Mean 6 MW distance was 418.8 (SD = 152.5) m, and average 6 MW speed was 69.8 (SD = 25.4) m/min, resulting in an average net O_2_ cost of walking of 0.16 (SD = 0.07) mL/kg/m; this value is similar to O_2_ costs of walking reported in other samples of persons with MS (i.e., 0.13–0.18 mL/kg/m) [6–8].

### 3.3. Associations among Disability, Symptoms, Gait, and Oxygen Cost of Walking

**Figure 2:**
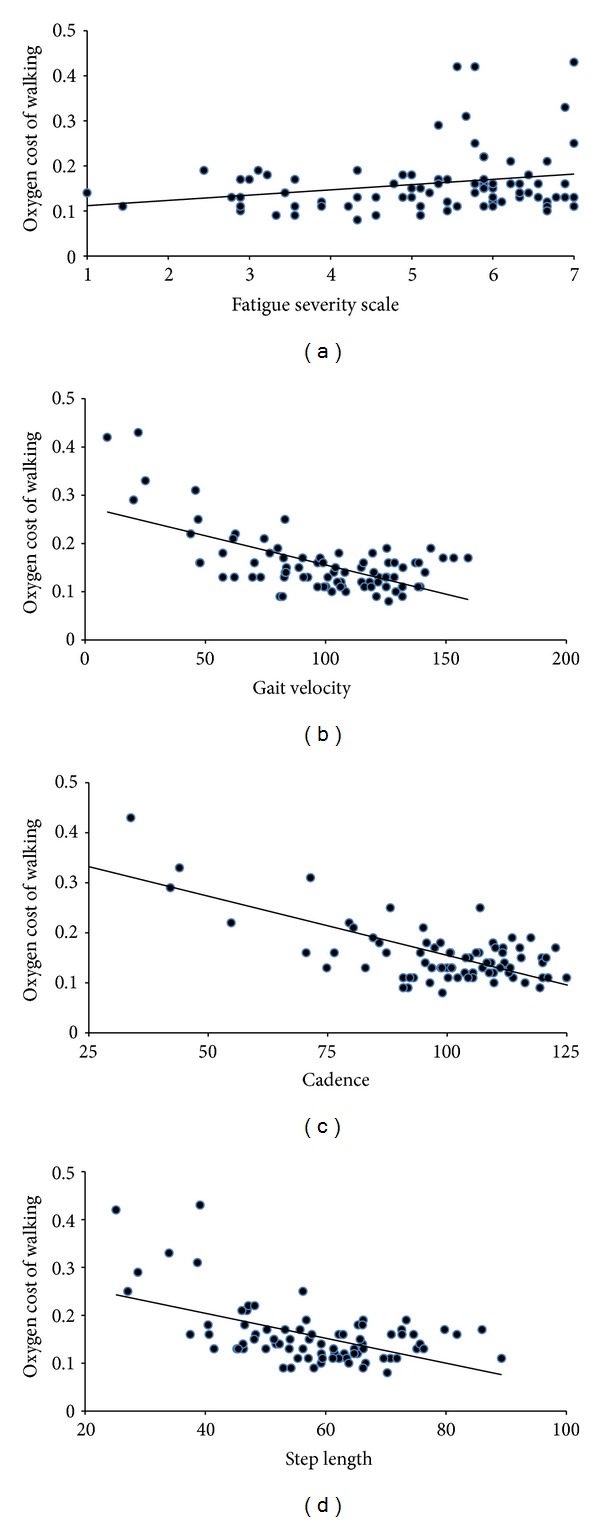
Scatter plots of oxygen cost of walking (mL/kg/m) and (a) fatigue, (b) gait velocity (cm/s), (c) cadence (steps/min), and (d) step length (cm) along with lines of best fit and 95% confidence intervals in 82 persons with multiple sclerosis.

 The correlations among variables for the overall sample are provided in Table 2. Briefly, PDDS scores were significantly associated with the O_2_ cost of walking (*r* = 0.546, *p* < 0.001) indicating that those with worse disability had worse walking efficiency. Regarding symptoms, fatigue, pain, and depression, but not anxiety, were significantly associated with PDDS scores. However, among symptomatic variables, only fatigue was associated with O_2_ cost of walking (*r* = 0.223); the scatter plot for this correlation is presented in Figure 2. Regarding spatiotemporal gait parameters, velocity, cadence, and step length were negatively associated with both PDDS scores and the O_2_ cost of walking, and the correlations were large in magnitude (*r*'s between −0.528 and −0.730). The scatter plots for these associations are further presented in Figure 2. Collectively, this indicates that persons with MS who had worse disability, fatigue, and gait (indexed by slower gait, characterized by fewer and shorter steps) expended more energy per meter walked.

### 3.4. Regression Analyses

The results for the hierarchical linear regression analyses are in Table 3. We included fatigue severity as the only symptomatic outcome in the regressions because this was the sole symptomatic variable that was significantly associated with both PDDS scores and O_2_ cost of walking. Disability status (*B* = 0.019, SE  *B* = 0.004, and *β* = 0.518) explained a statistically significant (*F*(1,79) = 28.959, *p* < 0.001) portion of variance in the O_2_ cost of walking (*R* ^2^ = 0.26) in Step 1. We included fatigue and gait velocity only, as velocity was strongly correlated with cadence and step length (i.e., cadence and step length are subcomponents of gait velocity) in Step 2a of the model. The **β*-*coefficient for disability status became attenuated and nonsignificant (*β* = 0.12, *p* = 0.43) and gait velocity (*β* = −0.57), but not fatigue (*β* = −0.10), explained a statistically significant amount of variance in the O_2_ cost of walking (Δ*R* ^2^ = 0.11). We were interested in the independent contributions of the subcomponents of gait velocity in Step 2b of the model and included cadence and step length in place of velocity. The **β*-*coefficient for disability status again became attenuated and nonsignificant (*β* = −0.04, *p* = 0.75), and cadence (*β* = −0.67, *p* < 0.001), but not step length (*β* = −0.14, *p* = 0.24), explained a statistically significant amount of variance in the O_2_ cost of walking (Δ*R* ^2^ = 0.26).

## 4. Discussion

This study examined symptoms and gait parameters as variables that explain the association between disability status with O_2_ cost of walking in 82 persons with MS who had a large range of mobility disability from ambulatory without assistance through bilateral support. O_2_ cost of walking was associated with disability status such that those with worse disability had a greater O_2_ cost of walking. Fatigue, gait velocity, cadence, and step length, but not pain, anxiety, and depression, were associated with both disability status and O_2_ cost of walking. Gait velocity, but not fatigue, explained the association between disability status and O_2_ cost of walking, and we further identified cadence as the primary component of gait velocity that explained the association. Collectively, such results provide further evidence that worse disability status is associated with higher O_2_ cost of walking and identify gait cadence as a target of rehabilitative interventions for possibly improving metabolic efficiency during walking in those with MS.

We have previously reported that the O_2_ cost of treadmill and over-ground walking is associated with disability status in persons with MS, and that spatiotemporal parameters of gait and fatigue are associated with the O_2_ cost of treadmill walking in persons with mild MS disability [6, 7]. The current study replicated and extended those findings using over-ground walking in a substantially larger sample of persons with MS who had a broader range of disability. The novel aspect of this study was that we included symptomatic and gait-related outcomes as potential mediators of the association between disability status and the O_2_ cost of walking in persons with MS. The primary novel findings were that fatigue and parameters of gait, but not other symptoms, were associated with disability and the O_2_ cost of walking and that cadence, but not step length, explained the association between disability status and O_2_ cost of walking. This indicates that cadence is the primary gait parameter that results in the energetic penalty of walking imposed by disability in MS.

To our knowledge, this study is the first to examine step length and cadence as intermediate variables in the association between MS disability and the O_2_ cost of walking. Interestingly, stride length (i.e., the sum of two step lengths) and cadence have been previously identified as partial mediators of the association between MS disability and 6 MW distance [23]. The current results suggest that cadence, but not step length, is a mediator of the association between MS disability and the O_2_ cost of walking during a 6 MW. Such results provide evidence for the general contributions of step length and cadence towards 6 MW distance and the specific contribution of cadence towards the O_2_ cost of walking. The identification of cadence as a significant mediator of the association between disability status and O_2_ cost of walking might be attributed to biomechanical inefficiencies during walking. Slower cadence is associated with increases in mediolateral displacement of the center of mass (i.e., poor biomechanical efficiency) during walking in healthy adults [28] and in persons with Down syndrome [29], presumably resulting in energetic penalties during walking (i.e., increases in the O_2_ cost of walking) [5]. As such, the current results support the development and implementation of interventions for improving gait cadence to minimize possible biomechanically induced increases in the O_2_ cost of walking that are associated with worsening MS disability. This might be accomplished through rhythmic auditory stimulation (RAS), as one pilot study reported large improvements (*d* = 0.70) in cadence following a one-week RAS intervention [30].

Pain, anxiety, and depression were not significantly associated with the O_2_ cost of walking. This was somewhat expected as previous research has indicated that those variables were not associated with 6 MW performance [31], although this had not been established for O_2_ cost of walking in persons with MS. There has been mixed evidence regarding the association between the O_2_ cost of walking and fatigue in samples of persons with mild MS during treadmill [7] and over-ground [8] walking tasks. Based on the current sample's broader range of disability, we did expect that fatigue would account, in part, for disability-related increases in O_2_ cost of walking. Although fatigue was significantly associated with both disability status and O_2_ cost of walking, fatigue did not explain the association between these variables; gait velocity, and ultimately cadence, emerged as a significant mediator of this association. This is reasonable as, presumably, persons with worse MS disability walk slower and expend more energy while walking, which might result in increased perceptions of fatigue. As such, future research might consider fatigue as a potential confound of the association between disability status and O_2_ cost of walking.

Another interesting aspect of the current study is that we provide further validity evidence for the PDDS as a measure of mobility disability in MS. Indeed, the PDDS has previously been validated as a measure of disability status and ambulatory status based on similarly strong associations with the EDSS and MSWS-12, T25FW, and 6 MW distance (**ρ**'s >0.63) [13]. The current study extends such validity evidence based on strong associations among the PDDS and O_2_ cost of walking (*r* = 0.55), gait velocity (*r* = −0.79), cadence (*r* = −0.68), and step length (*r* = −0.72). Collectively, this strengthens the body of evidence supporting the PDDS as a self-reported measure of disability status, particularly mobility disability, in MS.

Strengths of the current study include the relatively large sample size, inclusion of persons with a broad range of disability, and use of over-ground walking. This study is not without limitations. One limitation includes the cross-sectional research design, as it does not indicate causality between disability, gait, and O_2_ cost of walking. Nevertheless, we provide preliminary data necessary before investing considerable time, effort, and resources into designing and testing a rehabilitation program for improving cadence to minimize the disability-related O_2_ cost of walking in persons with MS. The current study did not include a comparison sample of healthy controls matched by age, sex, height, and weight in order to better examine the specificity of cadence as a predictor of the O_2_ cost of walking in persons with MS. Another limitation includes the use of the FSS as a self-report measure of fatigue. The FSS does not discriminate between physical and cognitive fatigue and might not be sufficient for identifying specific domains of fatigue as separate predictors of the O_2_ cost of walking in persons with MS. As such, future studies should consider the use of multidimensional scales (e.g., Fatigue Scale for Motor and Cognitive Functions [32]) for examining physical and cognitive fatigue as separate predictors of the O_2_ cost of walking in MS. One final limitation of the current investigation is that the gait parameters were not collected under the same walking conditions as the 6 MW. This could have provided additional insight to examine condition-related specificity of the associations among gait parameters and O_2_ cost of walking, though this was not an aim of this study.

## 5. Conclusions

This cross-sectional study examined symptoms and gait parameters as variables that account for the association between disability and the O_2_ cost of walking in 82 persons with MS. To that end, the primary novel findings were that fatigue and gait parameters, but not pain, anxiety, and depression, were associated with disability status and O_2_ cost of walking. Hierarchical linear regression analyses indicated that gait velocity, but not fatigue, explained variance in the association between disability status and O_2_ cost of walking. When decomposing velocity into its subcomponents of cadence and step length, only cadence accounted for the association between disability and O_2_ cost of walking. Collectively, these results suggest that slower cadence, perhaps due to biomechanical inefficiencies, is responsible for increases in O_2_ cost of walking among persons with advancing MS disability. This highlights the importance of designing and implementing interventions for minimizing gait-related energetic penalties during walking among persons with MS.

## Figures and Tables

**Table 1:** Symptomatic and gait characteristics for 82 persons with MS.

Variable	MS (*n* = 82)
FSS	5.18 (1.39)
SF-MPQ	10.18 (7.66)
HADS-anxiety	7.30 (2.41)
HADS-depression	7.80 (2.76)
Gait velocity (cm/s)	98.32 (33.00)
Cadence (steps/min)	98.97 (19.95)
Step length (cm)	58.00 (13.05)

*Note.* FSS: Fatigue Severity Scale; SF-MPQ: Short-Form McGill Pain Questionnaire; HADS: Hospital Anxiety and Depression Scale. All data are presented as mean (SD).

**Table 2:** Pearson product-moment correlations (*r*) among oxygen cost of walking, Patient-Determined Disease Steps scores, and symptomatic and gait variables in 82 persons with MS.

Variable	1	2	3	4	5	6	7	8	9
(1) O_2_ cost of walking	—								
(2) PDDS	0.546**	—							
(3) FSS	0.223*	0.539**	—						
(4) SF-MPQ	0.059	0.380**	0.515**	—					
(5) HADS-anxiety	−0.051	0.069	0.320**	0.279*	—				
(6) HADS-depression	0.078	0.291**	0.475**	0.401**	0.413**	—			
(7) Gait velocity	−0.620**	−0.791**	−0.457**	−0.358**	−0.082	−0.236*	—		
(8) Cadence	−0.730**	−0.684**	−0.318**	−0.234*	−0.017	−0.141	0.845**	—	
(9) Step length	−0.528**	−0.719**	−0.441**	−0.363**	−0.113	−0.229*	0.933**	0.626**	—

Note.**significance at *p* < 0.01; *significance at *p* < 0.05; PDDS: Patient-Determined Disease Steps; FSS: Fatigue Severity Scale; SF-MPQ: Short-Form McGill Pain Questionnaire; HADS: Hospital Anxiety and Depression Scale.

**Table 3:** Summary of hierarchical linear regression analyses for predicting oxygen-cost of walking in 82 persons with MS.

Variable	*B*	SE *B*	*β*
Step 1			
PDDS	0.0188	0.0035	0.518*
Step 2a			
PDDS	0.0045	0.0056	0.123
FSS	−0.0047	0.0048	−0.103
Gait velocity	−0.0011	0.0003	−0.570*
Step 2b			
PDDS	−0.0015	0.0045	−0.040
Cadence	−0.0022	0.0004	−0.671*
Step length	−0.0007	0.0006	−0.137

*Note. R* ^2^ = 0.259 for Step 1; Δ*R* ^2^ = 0.111 for Step 2a; Δ*R* ^2^ = 0.264 for Step 2b (*P* < 0.05, two-tailed test).

*P < 0.05 with one-tailed test.
